# Supplementary material for: Emulsifiers Impact Colonic Length in Mice and Emulsifier Restriction is Feasible in People with Crohn’s Disease
Source: Nutrients. 2020 Sep 15;12(9):2827. doi: 10.3390/nu12092827 (PMC7551245; doi:10.3390/nu12092827)
Supplement: Supplementary file 1 [file nutrients-12-02827-s001.pdf]

Supplementary Table S1: Excluded food additives on the low emulsifier diet

Food additive emulsifier	E number
Lecithin	E322
Sodium phosphates	E339i-iii
Alginic acid	E400
Sodium alginate	E401
Potassium alginate	E402
Ammonium alginate	E403
Calcium alginate	E404
Propylene glycol alginate	E405
Agar	E406
Carrageenan	E407
Processed euchema seaweed	E407a
Carob bean gum	E410
Guar gum	E412
Tragacanth gum	E413
Gum arabic	E414
Xanthan gum	E415
Karaya gum	E416
Tara gum	E417
Octenyl succinic acid modified gum arabic	E423
Konjac flour	E425
Soybean hemicellulose	E426
Cassia gum	E427
Polyoxyethylene (20) sorbitan monolaurate (polysorbate 20)	E432
Polyoxyethylene (20) sorbitan monooleate (polysorbate 80)	E433
Polyoxyethylene (20) sorbitan monopalmitate (polysorbate 40)	E434
Polyoxyethylene (20) sorbitan monostearate (polysorbate 60)	E435
Polyoxyethylene (20) sorbitan tristearate (polysorbate 65)	E436
Pectins	E440
Ammonium salts of phosphatidic acid	E442
Glycerol esters of rosin	E445

**Food additive emulsifier**	**E number**
Diphosphates	E450(i-viii)
Polyphosphates	E452(i-vi)
Celluloses	E460(i-ii)
Methyl cellulose	E461
Ethyl cellulose	E462
Hydroxypropyl cellulose	E463
Hydroxypropyl methyl cellulose	E464
Methyl ethyl cellulose	E465
Sodium carboxymethyl cellulose	E466
Ethyl hydroxyethyl cellulose	E467
Cross-linked sodium carboxymethylcellulose	E468
Sodium carboxymethyl cellulose enzymatically hydrolysed	E469
Salts of fatty acids, with base of Ca, Mg, K, Na, Al, NH_3	E470(i-iii)
Mono- and di glycerides of fatty acids	E471
Acetic and fatty acid esters of glycerol	E472a
Lactic and fatty acid esters of glycerol	E472b
Citric and fatty acid esters of glycerol	E472c
Tartaric acid esters of mono- and di-glycerides of fatty acids	E472d
Diacetyltartaric and fatty acid esters of glycerol	E472e
Mixed acetic and tartaric acid esters of mono- and diglycerides of fatty acids	E472f
Sucrose esters of fatty acids	E473
Sucroglycerides	E474
Polyglycerol esters of fatty acids	E475
Polyglycerol esters of interesterified ricinoleic acid	E476
Propylene glycol esters of fatty acids	E477
Lactylated fatty acid esters of glycerol and propylene glycol	E478
Thermally oxidised soya bean oil interacted with mono- and diglycerides of fatty acids	E479
Sodium lactylates	E481(i-ii)
Calcium lactylates	E482(i-ii)

Food additive emulsifier	E number
Sorbitan monostearate	E491
Sorbitan tristearate	E492
Sorbitan monolaurate	E493
Sorbitan monooleate	E494
Sorbitan monopalmitate	E495
Quillaia extracts	E999(i-ii)

Supplementary Table S2: Feasibility and acceptability of the low emulsifier diet in 20 people with stable Crohn's disease

**Question**	Response, n (%)			
	No	Slightly more	More	Much more
Meal preparation was more difficult	11 (55)	6 (30)	3 (15)	0 (0)
Longer time spent preparing and cooking meals	7 (35)	11 (55)	2 (10)	0 (0)
Longer time spent food shopping	0 (0)	8 (40)	8 (40)	3 (15)
Finding suitable foods when shopping was more difficult	2 (10)	11 (55)	6 (30)	1 (5)
Finding suitable foods when eating out was more difficult	6 (30)	1 (5)	3 (15)	10 (50)
The flavour of meals and snacks was less appetising	19 (95)	1 (5)	0 (0)	0 (0)
More money spent on food shopping and eating out	5 (25)	8 (40)	2 (10)	0 (0)
The diet was more difficult	2 (10)	14 (70)	4 (20)	0 (0)
Following the diet for 6-8 weeks would be more difficult than normal	2 (10)	8 (40)	8 (40)	1 (5)
